# Robot-assisted general surgery is safe during the learning curve: a 5-year Australian experience

**DOI:** 10.1007/s11701-023-01560-8

**Published:** 2023-03-10

**Authors:** Silas Nann, Abdul Rana, Alex Karatassas, Jimmy Eteuati, Darren Tonkin, Christopher McDonald

**Affiliations:** 1 University of Adelaide, North Terrace, Adelaide, South Australia 5000 Australia; 2 The Lyell McEwin Hospital, South Australia Elizabeth Vale, Adelaide, Australia; 3 Royal Adelaide Hospital, Adelaide, South Australia Australia; 4 The Queen Elizabeth Hospital, South Australia Woodville South, Adelaide, Australia

**Keywords:** Robotic surgery, Colorectal surgery, General surgery

## Abstract

Robot-assisted general surgery has become increasingly common in the Australian public sector since 2003. It provides significant technical advantages compared to laparoscopic surgery. Currently, it is estimated that the learning curve for surgeons starting off with robotic surgery is complete after 15 cases. This is a retrospective case series, following the progress of four surgeons with minimal robotic experience over 5 years. Patients undergoing colorectal procedures and hernia repairs were included. 303 robotic cases were included in this study, 193 colorectal surgeries and 110 hernia repairs. 20.2% of colorectal patients experienced an adverse event and 10.0% of hernia patients had a complication. The learning curve was correlated to the average docking time, and it was found that this was complete after 2 years, or after a minimum of 12 to 15 cases. Patient length of stay decreases as surgeon experience increases. Robotic surgery is a safe approach to colorectal surgery and hernia repairs with some potential benefits in terms of patient outcomes as surgeon experience increases.

## Introduction

Robotic surgery was first introduced to Australia in 2003. Since then, its use has become increasingly common [1]. Although currently mostly restricted to the private sector, surgeons have continued to consider the utility of the da Vinci robotic system in public health care. Currently, more widespread application is limited by the increased price tag, compared to laparoscopic surgery [2]. Robots have been theorised to provide technical advantages, particularly in anatomically challenging body compartments, like pelvis. This is because of a three-dimensional image and improved dexterity of surgical instruments, compared to laparoscopic surgery [3]. In most studies, robotic surgery has been shown to have comparative clinical outcomes, compared to laparoscopic surgery [4–7]. In fact, use of robots has been shown to reduce post-operative recovery time of bowel function and length of inpatient stay [4], decreased rates of conversion to open [6], and reduced peri-operative complications [8]. Although data regarding robotic surgery is still limited, it has been proven to be safe and effective.

Minimal information is available regarding the learning curve with robotic abdominal surgery. It has been shown that for experienced laparoscopic surgeons, the robotic learning curve is reduced. In a study by Odermatt et al. it was found that after 15 cases, all quality indicators were on target. [9] Shaw et al. has also found that after 15 robotic colorectal cases, complication rates are reduced to a baseline level [10]. There is some evidence that patient outcomes are improved with increased experience. Bastawrous et al. suggest that a surgeon who accomplishes 30 or more robotic colorectal cases per year will have improved performance in terms of operative time, length of stay, conversion rates, and direct total cost. No improvement in complication rates or readmission rates were demonstrated [11]. Although some authors conclude that an inexperienced robotic surgeon may have higher rates of adverse patient events, very little information is currently available regarding the learning curve and patient outcomes with robotic abdominal surgery.

In this study, we aim to analyse the performance of four surgeons performing robotic colorectal procedures and hernia repairs over a 5-year period in terms of docking time, surgical time, post-operative length of stay, and complication rates. We hypothesise that all measured parameters will improve, as surgeon experience increases.

## Methods

This is a retrospective case series of robotic colorectal procedures and hernia repairs over a 5-year period. Cases included are all performed by four surgeons (DT, AK, JE, ML) with no robotic surgery experience at two major private hospitals in Adelaide from the 9th May 2015 to 9th August 2019. A “da Vinci Surgical System” was used for all procedures recorded. To overcome the learning curve, a collaborative team approach was used for the initial 2 years of this study, whereby 82.8% of surgical cases were assisted by one of the other three surgeons. Patient demographics and surgical data were collected, including age and gender, ASA, BMI, docking time, surgical time, post-operative length of stay, and adverse events. Data were analysed by type of procedure (hernia and colorectal) and as a cohort with all procedures included.

Research was performed in accordance with relevant guidelines and regulations. Patient data were stored and managed in accordance with the Australian Code for the Responsible Conduct of Research. Research ethics approval was sought from and granted by a local HREC. The authors have no conflict of interest to disclose, although all senior authors are proctors for device technologies. The authors did not receive support from any organisation for the submitted work. The authors received no financial support for the research, authorship, and/or publication of this article.

## Results

### Patient demographic

This study included 303 patients, undergoing various robotic colorectal procedures and hernia repairs. A detailed summary of patient demographics is included in Table 1. The mean age of this cohort was 63 with a male to female ration of 1–1.52. Most patients, according to the ASA classification [12], pre-morbidly suffered from either a mild or severe, but not incapacitating systemic disease. This is reflected by the average ASA score of 2.35. The average BMI was 28.7. The highest recorded BMI of 63 was a female patient undergoing a high anterior resection.

**Table 1:** Patient demographics

	No. of participants (*n* = 303)
*Age*	
Mean age	63
Age range	19–91
*Gender*	
Male	120
Female	182
Ratio M:F	1:1.52
*Patient co-morbidity*	
Average ASA	2.35
Average BMI	28.7
BMI range	16 to 63

### Colorectal cohort

A variety of colorectal surgeries were successfully completed using a robotic approach. A detailed summary of all operations is provided in Table 2. The most common colorectal procedures performed include ventral mesh rectopexy, high anterior resection, hemicolectomy (either right or left), and ultralow anterior resection. Other procedures were also included, but performed less commonly.

**Table 2:** List of colorectal procedures performed

Type of procedure	Number of patients (*n* = 193)
Ventral mesh rectopexy	54
High anterior resection	47
Hemicolectomy	30
Ultralow anterior resection	20
Reversal of Hartmann’s	9
Low anterior resection	7
Abdominoperineal resection	6
Proctocolectomy	4
Ileocolic resection	3
Reversal of ileostomy	3
Hartmann’s procedure	2
Total colectomy	1
Other	7

Table 3 provides peri-operative data by year, as surgeon robotic experience increases. The average patient age remains relatively constant. The mean docking time was highest in 2015 with an average of 10.6 min. This decreases to an average between 5 and 6 min by 2017 and then remains largely unchanged. There is no decrease in average surgical time as surgeon experience increases and average length of stay is relatively constant between years.

**Table 3:** Performance data related to colorectal procedures over time

Year	Mean age	Mean docking time (mins)	Mean surgical time (mins)	Mean length of stay (days)	Complication all/major	Complication (%) all/major
2015 (*n* = 20)	66.3	10.6	220.1	4.5	5/0	25/0
2016 (*n* = 51)	62.4	7.3	206.5	5.7	13/5	25.5/9.8
2017 (*n* = 42)	60.7	6.1	200.8	4.4	10/3	23.8/7.1
2018 (*n* = 49)	65	5.4	233	6.2	9/4	18.4/8.2
2019 (*n* = 31)	66.1	5.5	206.2	4.7	2/1	6.5/3.2
Average	63.7	6.6	213.1	5.3		20.2/6.7

Complications were graded using the Clavien–Dindo system [13]. Major complications were defined by a classification grade of IIIa or higher. Of the 13 major complications listed, 5 cases required a conversion to open laparotomy because of intra-operative technical difficulties and patient factors. Reasons included difficulty with access to the pathology, unexpected anatomy (a lymph node in close proximity to the superior mesenteric vein), and to gain length for the anastomosis. Seven patients underwent unexpected returns to theatre because of anastomotic bleeding and small bowel obstructions in the recovery period. In 2017, a patient became febrile on day 3 after their operation and underwent a re-look laparotomy. A haematoma was evacuated. This patient required intensive care support post-operatively. One case was complicated by the needle snapping below the anastomosis. A variety of minor adverse events were also recoded and included portside bleeding/haematomas, ileus, wound infection, and re-admissions for pain management.

It was noted that the percentage of complications in general decreases over time. In 2015, a quarter of all cases were affected by intra-operative or post-operative adverse events. Similar percentages were recorded for the following 2 years, with 25.5% and 23.8%, respectively. This number steadily decreases year by year. In 2018, only 18.4% of cases were affected by any complication. In 2019, this decreased even further to 6.5%. The highest rate of major complications occurred in 2016 at 9.8%. This decreased to 7.1% in 2017 and was lowest in 2019 at 3.2%. A higher rate was again found in 2018 at 8.2%. It should be noted that of the four major complications that occurred in this year, two cases included a conversion to open laparotomy due to unexpected intra-operative patient factors. Because of the size of the lesion requiring resection in one instance and difficulties passing sizers into the abdomen in the other, a robotic approach was abandoned. If these cases were not listed as a complication, the rate in 2018 would be in line with the general trend at 4.1%.

### Hernia cohort

A variety of hernia surgery was successfully completed using a robotic approach. A detailed summary of all operations is provided in Table 4. The most common robotic hernia repairs performed include incisional hernia repairs and parastomal hernia repairs. Other procedures were also included, but performed less commonly.

**Table 4:** List of hernia procedures performed

Type of procedure	Number of patients (*n* = 110)
Incisional hernia repair	76
Parastomal hernia repair	14
Ventral hernia repair	9
Inguinal hernia repair	6
Spigelian hernia repair	5

Table 5 contains data relating to the peri-operative care of robotic hernia repair patients. The average age of this cohort is relatively consistent, with an overall mean of 62 years. Mean docking time is highest in 2015 and 2016. The one case in 2015 took 15 min to dock. In 2016, the average time required reduced to 7.5 min. After this year, mean docking time only decreased by minute amounts, with an average of 4.4 min per case in 2019. Mean surgical time did not decrease as surgeon experience increased. Average length of stay was highest in 2016. This also consistently decreased. In 2019, the average length of stay post-operatively was 2.4 days.

**Table 5:** Performance data related to hernia procedures over time

Year	Mean age	Mean docking time (mins)	Mean surgical time (mins)	Mean length of stay (days)	Complication all/major	Complication (%) all/major
2015 (*n* = 1)	44	13	67	2	1/0	100/0
2016 (*n* = 25)	59.9	7.5	197.1	4.1	3/3	12/12
2017 (*n* = 25)	63.8	5.2	161.9	2.8	5/2	20/8
2018 (*n* = 42)	62.6	4.8	193.5	2.8	1/0	2.3/0
2019 (*n* = 17)	62.1	4.4	181.1	2.4	1/0	5.8/0
Average	62.0	5.6	184.2	3.1	11/5	10/4.5

In terms of complications, it is noticeable that most major complications occurred early in the surgeon’s journey. Three major complications were logged in 2016 and one in 2017. Higher rates of all complications were noted early on as well. The highest rate of adverse events occurred in 2017. In this year, 20% of cases were affected by a complication. This decreased to 2.3% in 2018 and 5.8% in 2019.

Of the five major complications listed, two patients required unplanned post-operative intensive care admissions. One case was complicated by a PE and required warfarin therapy. The other patient developed aspiration pneumonia following extubation and was transferred to ICU for ventilatory support. Three cases were converted to an open laparotomy. In two instances, this was because of inadvertent small bowel injury, requiring bowel resection. The other case was complicated by camera malfunction, therefore requiring open surgery. A variety of minor adverse events were recorded, including wound site infection, readmission for pain management, and post-operative ileus.

### Length of stay (total cohort)

Table 6 displays the average length of stay of the entire cohort of 303 patients by year. The year with the longest average length of stay was 2016, with a mean of 3.2 days. Every year thereafter demonstrates a steady decline in the average length of stay. The year with the shortest average is 2019, with a mean of 1.67 days.

**Table 6:** Patient length of stay by year of total cohort

Year	Average length of stay (days)	Range (days)
2015 (*n* = 21)	2.4	1–5
2016 (*n* = 76)	3.2	2–6
2017 (*n* = 67)	3	1–6
2018 (*n* = 91)	2.7	1–6
2019 (*n* = 48)	1.67	1–2
Average	2.89	1–6

## Discussion

A large cohort of robotic surgery patients were included in this study. 193 patients underwent various colorectal procedures and 110 patients had various hernias repaired. These two cohorts are similar in terms of baseline patient characteristics; however, they behave differently when analysing peri-operative data. This is not unexpected, since colorectal surgery involves higher risk, more invasive, and longer procedures.

In this study, it is shown that robotic colorectal surgery is a safe intervention for patients, even when the primary surgeon has no robotic experience. The overall rate of adverse events was 20.2% with a major complication rate of 6.7%. A major complication was defined as a Clavien–Dindo classification of grade III or above. This is comparable to other publications that estimate the overall complication rate between 16.3and 17.6% [14, 15]. Laparoscopic colorectal surgery has been shown to have morbidity in 20.1% of cases in a large, multicentre trial by Rose et al. [16]. It should be noted that complication rates decrease over time. The highest rate was reported in 2015 with 25%. This steadily decreases to 6.5% by 2019. Our findings therefore suggest that robotic colorectal surgery is safe, compared to the more commonly performed laparoscopic approach and that complication rates may be even lower than this in the hands of an experienced surgeon. Rates of major complications are acceptably low at all points of the learning curve.

Less literature is available regarding robotic hernia repairs. This study demonstrates that this is a safe technique with results that are potentially superior to the current standard of care. Currently, most abdominal and groin hernias are repaired with an open approach. In this cohort, 10% of patients experienced any complication during their admission, with a major complication rate of only 4.5%. Other studies, examining more commonly used surgical techniques, have found higher rates of adverse events for similar procedures. The rate of complications with laparoscopic ventral and incisional hernia repair was reported between 8 and 13% [17, 18]. Open ventral hernia repair has been associated with complication rates of 21% [18]. Elective open groin hernia surgery has been shown to have adverse events in 15.1% of instances [19]. In comparison with these figures, robotic surgery has lower complication rates, on average. This is despite surgeon inexperience early on in this case series.

Mean docking time provides the most consistent indication of the surgeon’s learning curve, since the docking process is similar between different robotic procedures. Both the colorectal and the hernia cohorts suggest that the learning curve for robotic surgeons is approximately 2 years. After 2 years, no further improvements in this time were achieved. In the first 2 years, AK had performed 12, JE 24, CM 15, and DT 49 robotic procedures. Other studies have suggested that an experienced laparoscopic surgeon will complete their learning curve after 15 cases [9, 10]. This study is in keeping with this estimate. It appears that surgeons achieve a consistent docking time after 12–15 cases.

No improvements were found in surgical time as surgeon experience increased for both cohorts. Two factors account for this: case complexity and surgical technique. Once a surgeon has completed their learning curve, the complexity of cases performed using a robotic approach also increases, which adds to the operative time. Likewise, more sophisticated surgical techniques are utilised as surgeon confidence grows. An example of this in the presented cohort is the approach to ventral mesh repair. Prior to 2017, all cases were repaired using intraperitoneal onlay mesh (IPOM) only. After this time point, an enhanced-view totally extraperitoneal (eTEP) approach was increasingly used by surgeons. Although this technique added operative time, this was outweighed by benefits in terms of recovery time and post-operative pain [20]. Case complexity and surgical approach also explain why colorectal procedures are generally more time intensive than hernia repairs.

It is interesting to note that the average length of stay of the entire cohort tended to decrease over time. In 2016, the average post-operative length of stay was 3.2 days and this steadily decreased to 1.67 days by 2019, as surgeon experience increased. This trend is also demonstrated in the hernia cohort, where the longest average length of stay is reported in 2016 with an average of 4.1 days. This mean decreases in subsequent years to an average of 2.4 days in 2019. It is likely that improvements in surgical techniques results in a quicker recovery and therefore superior outcomes for patient. No improvements in terms of length of stay was demonstrated in the colorectal cohort. This observation is likely related to the increasing complexity of colorectal cases as surgeon confidence increased, requiring a lengthier post-operative recovery course.

A collaborative team approach to overcome the robotic learning curve has been shown to improve patient outcomes for urological procedures [21]. The authors have applied this strategy to robotic general surgery. During the first 2 years of practice with the da Vinci robot, all four surgeons were present in the theatre for almost every patient that was included in this study and 82.8% of cases were assisted by one of the other three surgeons. Although primary operator experience increased more slowly, all surgeons built experience as assistant or observer. If difficulty was encountered during the operation, this could be discussed in real time and all surgeons would learn from the experience. Patient safety was also improved since four experienced colorectal surgeons were present in person to provide expertise and input. The authors suggest that this approach should be considered by surgeons who want to train in robotic colorectal surgery.

The main limitation of this study is that this is a retrospective analysis. Generalisability is therefore limited. Future randomised trials are recommended.

## Conclusion

This study shows that robotic surgery is a safe approach for both hernia repairs and colorectal surgery. Complication rates are comparable to other surgical techniques. 20.2% of colorectal patients and 10.0% of hernia patients experienced adverse events. It was found that the learning curve for robotic surgery is complete at the 2-year mark, or with a minimum of 12–15 cases. It was also shown that length of stay reduces as surgeon experience increases in the hernia cohort, which indicates that superior patient outcomes are achieved over time.

## Data Availability

The data that support the findings of this study are available on request from the corresponding author, SN. The data are not publicly available due to their containing information that could compromise the privacy of patients.
